# Coconut Sugar: Chemical Analysis and Nutritional Profile; Health Impacts; Safety and Quality Control; Food Industry Applications

**DOI:** 10.3390/ijerph20043671

**Published:** 2023-02-19

**Authors:** Ariana Saraiva, Conrado Carrascosa, Fernando Ramos, Dele Raheem, Maria Lopes, António Raposo

**Affiliations:** 1 Department of Animal Pathology and Production, Bromatology and Food Technology, Faculty of Veterinary, Universidad de Las Palmas de Gran Canaria, Trasmontaña s/n, 35413 Arucas, Spain; 2 Faculty of Pharmacy, University of Coimbra, Azinhaga de Santa Comba, 3000-548 Coimbra, Portugal; 3 Associated Laboratory for Green Chemistry (LAQV) of the Network of Chemistry and Technology (REQUIMTE), Rua D. Manuel II, Apartado 55142, 4051-401 Porto, Portugal; 4 Northern Institute for Environmental and Minority Law (NIEM), Arctic Centre, University of Lapland, 96101 Rovaniemi, Finland; 5 CBIOS (Research Center for Biosciences and Health Technologies), Universidade Lusófona de Humanidades e Tecnologias, Campo Grande 376, 1749-024 Lisboa, Portugal

**Keywords:** alternative sweeteners, coconut sugar, chemical analysis, health impacts, nutrition, food industry

## Abstract

Consumers often wish to substitute refined sugar with alternative sweeteners, such as coconut sugar, given growing interest in healthy eating and the public’s negative perception of excess sugar intake. Coconut sugar is a healthier, sweetener option than the majority of other sugars that are commercially available. Sap is collected from trees to be transported, stored, and evaporated during processing, which are labor- and resource-intensive operations. Consequently, the cost of production is higher than it is for cane sugar. Given its high nutritional value and low glycemic index, people are willing to pay higher prices for it. However, one barrier is ignorance of its health benefits. This review examines and deals in-depth with the most significant features of coconut sugar chemical analyses to focus on several analytical methodologies given the increasing demand for naturally derived sweeteners in the last 10 years. A deeper understanding of the quality control, safety, health effects, nutritional profile, and sustainability issues corresponding to coconut sugar is necessary to effectively implement them in the food industry.

## 1. Introduction

In south/south-eastern Asian cuisine, coconut sugar is a popular sweetener [1] and is made of phloem sap from coconut palm tree (*Cocos nucifera* L.) blossoms [2]. Workers collect sap by scaling palm trees and use sickles to chop off unopened inflorescences. For 8–12 h, oozing sap is collected with bamboo or plastic containers. Lime is occasionally added to the sap to stop it from fermenting [3,4]. Next, the sap is heated on open flames and regularly shaken for it to thicken and crystallize [1]. During the production method, sugar color can range from light to dark brown. Finally, sugar is hand-selected and sieved to produce fine-grained produce [5].

One inflorescence is typically produced by each coconut palm tree once a month. Approximately 1.5 L of sap is harvested twice a day (morning and evening) from all the inflorescences. Based on the approximate 15 g of sugar per 100 g sugar content of fresh coconut sap, boiling sap allows 200 g of sugar per inflorescence to be produced daily [3,4].

Even at early ages, coconut palm trees can be utilized for sap collection purposes. Every time phloem sap is tapped and harvested, 1–2 mm of spadix must be cut away. Spadix can be diminished to a stump by repeating this technique. Following this procedure, a single spadix can be tapped for 40–45 days. Coconut palm trees can be tapped for a 20-year period [1,3].

Due to the growing interest that the public is showing in healthy diet and the negative public perception of excess sugar use, consumers frequently attempt to substitute refined sugars for alternative sweeteners like coconut sugar [6]. Traders highlight coconut sugar’s traditional small-farmer producers, organic palm tree growth in mixed farming with other crops, lower glycemic index (GI), and low fructose content than regular refined beet sugar or cane [7]. Coconut sugar has a premium price that consumers are willing to pay. One kilogram (kg) might cost something between €15 and €46. In contrast, the price of a kg of traditionally refined sugar was only €0.88 in 2021 [8].

Customers today are increasingly more aware of natural ingredients. Consumers’ growing emphasis on naturalness has had a significant impact on the food industry [9,10]. Consumers in most nations often reject the food products that they do not perceive as natural. The demand for sweeteners made from natural sources has skyrocketed in recent decades [11].

In light of the above, the present review investigates the health effects and nutritional profile linked with consuming coconut sugar, its potential food industry applications and sustainability issues, and its primary safety–quality parameters, plus a chemical analysis of its major components.

## 2. Chemical Analysis

Food attributes like color, consistency, texture, flavor, and smell are extremely relevant for consumers and, consequently, for industry. In coconut sugar and syrup, these characteristics derive from the quality of the sap from which they are produced and the chemical reactions that occur during the heating process, namely non-enzymatic browning via caramelization and Maillard reactions (MR) [12]. The latter involves a highly complex reaction between reducing sugars and amino acids, and has major effects on coconut sugar and syrup properties, including their nutritional and functional value, color, aroma, and flavor [12,13,14,15]. Regarding flavor, the characteristics of MR products may vary from a pleasant flowery aroma to a burnt aroma in accordance with the sugar and amino acid compositions of the food matrix and the involved reaction pathways [13,14,15,16]. MR products, such as acrylamide and 5-hydroxymethylfurfural, have been directly linked with the degree of coconut sugar browning, and high levels of these products can lead to toxic health effects [13,14,15,16,17]. In this context, it is worth noting the study by Phaenon et al. [18] in which the level of acrylamide was determined in both coconut sap and coconut syrup, and, while in the first case this compound was not detected, the reported levels in the latter were of 867 µg/kg. Many MR products exhibit beneficial biological functions, such as potent antioxidant activity [13]. As a matter of fact, the coconut sugar and syrup production process generates hundreds of distinct MR products that are more or less desirable [19]. Bearing this in mind, it is easy to understand that coconut sugar and syrup are much more than merely a concentrated sugar solution, and the study of their chemical composition, which is fundamental to improve their nutritional and functional properties and to guarantee consumer safety, is a complex and demanding task.

Several research works have been conducted to evaluate the physico-chemical, microbiological, and antioxidant characteristics of coconut sap, sugar, and syrup. Some of the most important ones are presented in Table 1 and focus on the analytical techniques used and the main outcomes. Color determination, pH, and total soluble solids are some of the analyses routinely performed in coconut sap, sugar, and syrup. However, despite being fundamental for the quality control of these products, they provide limited means for more specific quality profile analyses and, hence, the need for more advanced analytical techniques arises.

Coconut sugar and syrup contain well over 100 different types of compounds, including carbohydrates, free amino acids, proteins, minerals, vitamins, aromatic compounds, and phenolics. Table 2 provides an overview of some of the most recent studies carried out to assess the inorganic and organic compositions of coconut sap, sugar, and syrup. Atomic absorption spectroscopy (AAS) is the most commonly followed technique reported for mineral analyses. The main issue in determining mineral composition is the accurate quantification of these elements in a complex matrix, like that of coconut sugar or syrup, which has other components and at much higher levels (e.g., sugars), while dealing with the problem of interferences from mineral elements other than that being measured [20]. AAS is a technique with good detection limits. It is relatively simple to perform and incurs low to moderate acquisition and operation costs. It allows only a limited number of elements to be analyzed [19]. Other techniques like ICP-MS (inductively coupled plasma mass spectrometry) require costlier equipment and greater proficiency to operate, but stand out for having better detection limits and allow the quantification of many elements at ultra-trace concentrations in large numbers of samples. Therefore, they should be increasingly used [20]. For studying organic constituents, the non-volatile ones are analyzed mainly by high-performance and ultrahigh liquid chromatography (HPLC/UHPLC) coupled to different detectors (e.g., refractive index (RI), mass spectrometry (MS), UV-Vis), while volatile ones are determined mostly by GC–MS (gas chromatography coupled to mass spectroscopy) (e.g., [21,22,23]). Indeed, coupling a chromatography system (e.g., UHPLC/HPLC or GC) to a mass spectrometer is one of the most powerful ways to identify and quantify compounds because this analytical strategy provides two different types of data per run analysis: (i) retention time and (ii) mass spectral pattern (molecular ion and fragmentation), for each separated compound. This information can be used to make comparisons to appropriate reference standards or literature data [24].

As part of quality control, another critical aspect is the need to develop swift and accurate analytical methods for fraud detection. Like honey, agave syrup, and maple syrup, coconut sugar and syrup are prone to adulteration via the addition of less expensive exogenous sugars, such as cane sugar, beet sugar and corn sugar. In fact, the addition of a minor quantity of cane sugar, i.e., less than 5% *w*/*v*, to coconut sugar for “seed” purposes and to accelerate its crystallization is common and well-accepted in the industry [25]. The risk of fraud is a major issue given the important economic advantage of adding an extra amount of cane sugar or another inexpensive sugar [25]. Some of the most relevant analytical approaches recently proposed to combat this problem are presented in Table 3. Technologies like NMR (nuclear magnetic resonance) and IRMS (isotope ratio mass spectrometry) are noteworthy, as shown by the results obtained with the studies of Bachmann et al. [26] or Rogers et al. [25], respectively. NMR is a non-destructive technique that provides fast results and requires easy sample preparation. However, it implies using a large amount of sample to obtain an adequate signal [27]. IRMS, however, requires a considerably smaller amount of sample than in NMR analyses and displays 0.2‰ precision on the δ-scale [28]. One important limitation of IRMS is the fact that it only provides the global δ^13^C values of the analyte [28]. Specifically, in the coconut sugar and syrup adulteration context, applying the IRMS tool involves a significant disadvantage because, although it is highly successful for detecting the addition of cane and corn sugar to coconut sugar and syrup, it does not allow the detection of beet sugar addition [18,29]. This is because coconut is a C3 plant-like beet, while cane and corn are C4 plants. The ^13^C/^12^C ratio of C3 plants is lower than that of C4 plants, and it is on this difference that the IRMS technique is based because it is impossible to detect admixtures of beet sugar with coconut sugar [29] Notwithstanding, both IRMS and NMR have a high potential, and their combined approach could shed considerable light on the food fraud and adulteration issue. Energy-dispersive X-Ray fluorescence (ED-XRF) is an alternative tool with a very high potential for detecting coconut sugar adulterations with both cane and beet sugars, as evidenced by Zdiniakova and Calle [29]. ED-XRF offers the excellent advantage of requiring almost no sample preparation and can be performed with portable devices, but has relatively high quantification limits [29,30]. That said, from an analytical point of view and despite important advances, there is still a lot of work to be done to gain fast and eco-friendly methods that can be performed with portable devices and used by both consumers and producers quickly to easily detect adulterations.

In addition to food fraud, another aspect that cannot be neglected is food safety. Coconut sugar and syrup undoubtedly provide consumers with important nutritional and functional benefits when produced from high-quality sap that is properly collected, preserved, and processed, i.e., following good manufacturing practices (GMP). However, employing sap of inferior quality and its processing under unhygienic conditions are real problems that deserve our utmost attention because they pose the risk of contamination by insects and microorganisms (see Table 4). In this context, the dire need to carry out studies to determine the risk of other contaminants occurring is noteworthy. Research work on contaminants from food processing (acrylamide), agrochemicals (e.g., pesticide residues), heavy metals (e.g., mercury, lead, arsenic, cadmium, etc.), microorganism toxins (e.g., mycotoxins), and cleaning agents (e.g., detergents) or disinfectants (quaternary ammonium, detergents) has been increasingly performed in other natural sweeteners and sugar products, but is still almost nonexistent for coconut sugar and syrup.

**Table 1:** Physico-chemical, microbiological, and antioxidant characteristics of coconut sap, sugar, and syrup.

	Studies						
	Samples			Methodology	Analytical Details	Principal Outcomes	Ref.
	No.	Kind	Origin				
**Physico-Chemical Parameters**							
Color	2 *	Coconut sap (collected by two different methods)	India (Kasaragod)	Sensory analysis	A panel of 50 evaluators used a 5-point hedonic scale.	The authors assessed the color attributes of the sap collected by (1) the traditional approach using a lime-coated clay pot; (2) a novel “coconut-sap chiller method”. It was designed to collect fresh non-fermented sap free of foreign matter. The coconut sap collected by the traditional method was found to be oyster white, while that collected by the new method was golden brown.	[21]
	3 *	Coconut syrup (produced by three different methods)	Malaysia (Jelai)	Colorimetry	Outcomes were included in the CIELAB system: L* (lightness/darkness); a* (redness/greenness); b* (yellowness/blueness).	The authors investigated processing coconut sap in syrup by alternative processes compared to the conventional open heat evaporation technique. The L* parameter ranged from 23.84 to 35.61, a* from 2.31 to 3.746, and b* from 17.71 to 23.30.	[22]
	107 *	Coconut sugar	Indonesia, Philippines, and unknown origin	Sensory analysis	A descriptive test was carried out according to the official German methodology. For this purpose, a panel made up of 18 trained evaluators was selected.	The most expensive coconut sugars were light brown.	[17]
	6 *	Coconut sugar and coconut syrup	Philippines (Makati)	Colorimetry	Outcomes were included in the CIELAB system: L* (lightness/darkness); a* (redness/greenness); b* (yellowness/blueness).	The color of both coconut sugar and coconut syrup was evaluated for 6 months. For coconut sugar, the L* parameter ranged from 53.38 to 63.50, a* from 7.50 to 9.08, and b* from 24.02 to 28.28. For coconut syrup, L* ranged from 17.87 to 19.96, a* from 7.72 to 8.26, and b* from 1.44 to 1.67.	[31]
Consistency and texture	107 *	Coconut sugar	Indonesia, Philippines, and unknown origin	Sensory analysis	A descriptive test was carried out according to the official German methodology. To do so, a panel made up of 18 trained evaluators was selected.	The more expensive products, i.e., those that were light brown, were characterized as fine powders, while the cheaper ones were described as being coarse-grained.	[6]
Smell	2 *	Coconut sap (collected by two different methods)	India (Kasaragod)	Sensory analysis	A panel made up of 50 evaluators used a 5-point hedonic scale.	The sap collected by the traditional method had a fetid smell, which was not detected in the sap collected by a novel “coconut-sap chiller method” that the authors put forward. It is noteworthy in the traditional method that the collection system is open. This allows the emission of volatile molecules by sap to attract insects like bees, which leads to sap contamination. As the method developed by the authors is a closed system, this contamination does not occur.	[21]
	107 *	Coconut sugar	Indonesia, Philippines, and unknown origin	Sensory analysis	A descriptive test was applied in accordance with the official German methodology. For this purpose, a panel made up of 18 trained evaluators was selected.	In cheaper coconut sugars, i.e., those with a darker color, the sweet aroma predominated, while the caramel aroma was particularly dominant in the expensive products.	[6]
Flavor	2 *	Coconut sap (collected by two different methods)	India (Kasaragod)	Sensory analysis	A panel was made up of 50 evaluators who used a 5-point hedonic scale.	The flavor of the sap obtained by a novel “coconut-sap chiller method” was sweet and delicious, but that traditionally collected had a foul astringent aftertaste.	[21]
	107 *	Coconut sugar	Indonesia, Philippines, and unknown origin	Sensory analysis	A descriptive test was carried out according to the official German methodology. A panel made up of 18 trained evaluators was selected.	The coconut sugar flavor was described as mainly sweet. For the expensive products, i.e., those lighter in color, malty and caramel attributes were added.	[6]
pH	2 *	Coconut sap (collected by two different methods)	India (Kasaragod)	pH meter	NR **	The pH of the coconut sap collected by the traditional method was <6, whereas the sap collected by a new “coconut-sap chiller method” had a pH of 7–8. The sap collected by the novel method was fresh and non-fermented, but the traditionally collected sap was partially fermented.	[21]
	2 *	Coconut sap (with and without preservative, i.e., limestone solution)	Kemloko (Indonesia)	NR **	NR **	The pH levels of the fresh coconut sap, both with and without added preservative/s, were 4.26 and 4.68, respectively.	[32]
	6 *	Coconut sugar and coconut syrup	Philippines (Makati)	pH meter	NR **	The pH levels in coconut sugar and coconut syrup were evaluated over 6 months and ranged from 5.11 to 5.79 for the former, and from 4.28 to 4.45 for the latter. It should be noted that pH levels lowered in both products over time, which may be related to microbiological contamination.	[31]
Density	2 *	Coconut sap (collected by two different methods)	India (Kasaragod)	Refractometry	A refractometer measured both total soluble solids and Brix values.	The soluble solids content was higher in the sap collected by a new “coconut-sap chiller method” proposed by the authors (15.5–18 ^a^) compared to that determined in the sap collected by the conventional procedure (13–14 ^a^). This might be owing to the metabolization of sugars in sap by the microorganisms found in it, which were detected in much larger numbers in the conventionally collected sap. ^a^—Values expressed as °Brix.	[21]
	6 *	Coconut sugar and coconut syrup	Philippines (Makati)	Refractometry	A refractometer measured both total soluble solids and Brix values.	Brix was evaluated over 6 months for coconut sugar and syrup, and ranged from 97.6 to 98.9 for the former and from 79.6 to 80.3 for the latter.	[31]
**Microbiological parameters**	2 *	Coconut sap (fresh and 12-h fermented)	India (Kasaragod)	Metagenomic analysis	A culture-independent metagenomic methodology suitable for bacterial and fungal microbiome determinations with 16S rRNA and ITS amplicon sequencing, respectively, was used to perform the analysis of the fresh and fermented coconut sap.	The analysis of the microbiome of the fresh and fermented coconut sap revealed that the former presented a considerably larger number of bacterial species than the latter. In contrast, the fresh sap showed lower fungi and yeast diversity than the fermented sap. The fresh coconut sap displayed an abundance of *Leuconostoc* spp., followed by akin proportions of *Acetobacter* sp., *Fructobacillus* sp., and *Gluconobacter* sp. The fermented coconut sap exhibited a substantial increase in *Gluconobacter* sp. with a marked reduction in *Leuconostoc* spp. Regarding fungi and yeast occurrence, the fresh sap showed a predominance of species of the *Saccharomyces* genera and of *Hanseniaspora*. The fermented sap showed abundance for *Cortinarius saturatus* and *Hanseniaspora guilliermondii*.	[33]
**Antioxidant potential**	3 *	Coconut sap (collected by two different methods) and coconut sugar	India (Kasaragod)	Colorimetry	FRAP (ferric-reducing antioxidant power) assay.	According to the FRAP assay, the values of the conventionally collected coconut sap, the coconut sap obtained by a novel “coconut-sap chiller method” and the coconut sugar generated from the latter were respectively 8.34 ^a^, 14.8 ^a^, and 22.9 ^a^ ^a^—Values are expressed as mg of AEAC (ascorbic acid equivalent antioxidant capacity)	[21]
	2 *	Coconut sugar	Thailand (Samut Songkhram and Phetchaburi)	Colorimetry	The DPPH (2,2-Diphenyl-1-picrylhydrazyl) radical scavenging activity assay. The ORAC (oxygen radical antioxidant capacity) assay.	The DPPH free radical inhibition percentage and ORAC values ranged between 25.7–87.37 ^a^ and 740.7–3815.6 ^b^, respectively. ^a^—Values shown as %; ^b^—Values shown as mg of trolox equivalents (TE)/100 g.	[34]

* Total number of samples analyzed in the study; ** NR—Not reported.

**Table 2:** Chemical analyses of the organic and inorganic constituents of coconut sap, sugar, and syrup.

	Studies						
	Samples			Methodology	Analytical Details	Principal Outcomes	Ref.
	No.	Kind	Origin				
**Inorganic constituents**	9 *	Coconut sugar	Ivory Coast	Spectrometry	Sample preparation: Coconut sugar was incinerated until ash was obtained. Sample processing: The coconut sugar ash analysis was performed by (SEM).	The mineral levels in coconut sugar samples fell within the ranges of 101.77–128.95 ^a^ (K), 85.32–94.66 ^a^ (Cl), 7.96–16.28 ^a^ (Mg), 12.68–15.87 ^a^ (Si), 8.33–14.57 ^a^ (P), 5.58–13.17 ^a^ (S), 8.05–11.65 ^a^ (Na), 1.23–2.19 ^a^ (Cu), and 1.73–2.09 ^a^ (Fe). Traces—1.04 ^a^ (Br) and 0.17 ^a^ (Zn). ^a^—Values expressed as mg/100 g.	[35]
	1 *	Coconut sap	Malaysia (Jelai)	FAAS (flame atomic absorption spectrophotometry)	Sample preparation: Coconut sap was first diluted (10×), then filtered and further analyzed.	The predominant minerals were K (960.87 ^a^), Na (183.21 ^a^) and Mg (22.91 ^a^). The levels of Fe (1.36 ^a^), Ca (0.42 ^a^), Zn (0.338 ^a^), Mn (0.105 ^a^) and Cu (0.065 ^a^) were also determined. ^a^—Values expressed as mg/L.	[36]
	6 *	Coconut sugar and coconut syrup	Philippines (Makati)	Atomic absorption spectrophotometry (AAS)	NR **	The mineral composition of both coconut sugar and coconut syrup was evaluated for 6 months. For coconut sugar, the K, Na, and Fe ranges were 954–1075 ^a^, 99–112 ^a^, and 0.5–0.6 ^a^, respectively. The Ca and Zn levels remained constant over time and were 8 and 0.1 ^a^, respectively. For coconut syrup, the levels of K varied between 609–632 ^a^, Na between 110–126 ^a^, Ca between 1–2 ^a^, and Zn between 0.1–0.2 ^a^. The Fe levels were 0.4 ^a^ at the three different measurement times. ^a^—Values expressed as mg/100 g.	[31]
**Organic constituents**							
**Non volatiles**							
Amino acids	3 *	Coconut sap (collected by two different methods) and coconut sugar	India (Kasaragod)	Ninhydrin method (for free amino acids quantification) UHPLC-TQD-MS/MS (ultrahigh performance liquid chromatography coupled to tandem quadrupole mass spectrometry) for the amino acids profile analysis	Amino acids profile: Sample preparation: Samples were hydrolyzed in a vacuum using hydrochloric acid, 6 M. Then, hydrolysates were dried (also in a vacuum) after neutralization. They were dissolved in a known volume of the mobile phase, filtered (0.2 µm), and finally injected into the analytical system. Column: Waters UPLC BEH-C18 column (2.1 × 50 mm; 1.7 μm), protected by a Waters Vanguard BEH C-18 guard column (1.7 μm). Mobile phase: 0.1% formic acid in water-methanol: water (1:1) with 0.1% formic acid.	The total free amino acids content of the sap attained conventionally, the sap obtained from a “new coconut-sap chiller method”, and the sugar produced from the latter was, respectively 0.413 ^a^, 1.03 ^a^, and 3.05 ^b^. The following amino acids were quantified in the coconut sap obtained by the traditional method: (i) glutamic acid (359 ^c^); (ii) aspartic acid (83.7 ^c^); (iii) serine (60.5 ^c^); (iv) alanine (16.2 ^c^); (v) threonine (13.7 ^c^); (vi) proline (13.1 ^c^); (vii) arginine (11.9 ^c^); (viii) lysine (7.81 ^c^); (ix) valine (6.48 ^c^); (x) citrulline (6.38 ^c^); (xi) methionine (5.90 ^c^); (xii) phenylalanine (2.14 ^c^); (xiii) asparagine (0.86 ^c^); (xiv) leucine (0.64 ^c^); (xv) histidine (0.42 ^c^); (xvi) tyrosine (0.29 ^c^); (xvii) tryptophan (0.01 ^c^). For the sap acquired by the novel method, amino acids and respective levels were as follows: (i) glutamic acid (626 ^c^); (ii) aspartic acid (118 ^c^); (iii) serine (58.1 ^c^); (iv) arginine (17.3 ^c^); (v) alanine (15.1 ^c^); (vi) proline (14.6 ^c^); (vii) threonine (12.2 ^c^); (viii) methionine (6.28 ^c^); (ix) valine (6.10 ^c^); (x) citrulline (6.07 ^c^); (xi) lysine (5.93 ^c^); (xii) phenylalanine (2.56 ^c^); (xiii) asparagine (2.41 ^c^); (xiv) histidine (0.65 ^c^); (xv) leucine (0.56 ^c^); (xvi) tyrosine (0.16 ^c^); (xvii) tryptophan (0.01 ^c^). In turn, sugar contained (i) glutamic acid (394 ^d^); (ii) aspartic acid (131 ^d^); (iii) proline (112 ^d^); (iv) alanine (84.5 ^d^); (v) serine (78.0 ^d^); (vi) lysine (64.5 ^d^); (vii) threonine (59.1 ^d^); (viii) arginine (53.7 ^d^); (ix) valine (50.9 ^d^); (x) phenylalanine (50.7 ^d^); (xi) tyrosine (26.0 ^d^); (xii) leucine (21.8 ^d^); xiii) methionine (19.6 ^d^); (xiv) histidine (5.83 ^d^); (xv) citrulline (5.79 ^d^); (xvi) asparagine (4.25 ^d^); (xvii) 3,4-dihydroxy-phenylalanine (0.76 ^d^); (xviii) tryptophan (0.18 ^d^). ^a^—Values appear as g/100 mL; ^b^—Values appear as g/100 g; ^c^—Values appear as mg/100 mL. ^d^—Values appear as mg/100 g	[21]
Carbohydrates	3 *	Coconut syrup (produced by three different methods)	Malaysia (Jelai)	HPLC-RID (high-performance liquid chromatography coupled with refractive index detection)	Sample preparation: Coconut syrup samples were diluted (10×), filtered (0.45 µm), and further analyzed. Column: Merck LiChroCART^®^ Single bond NH_2_ column (250 × 4.6 mm; 5 µm). Mobile phase: Acetonitrile-water (80:20, *v*/*v*).	The authors investigated the processing of coconut sap in syrup by alternative process techniques compared to the conventional open heat evaporation technique. The fructose, glucose. and sucrose levels respectively ranged between 18.27–35.07 ^a^, 21.38–23.71 ^a^, and 7.35–25.67 ^a^. The coconut syrup obtained by the rotary evaporation technique displayed larger quantities of glucose and fructose, but a smaller quantity of sucrose, than that produced by the other techniques. The total sugar content for all the analyzed samples was between 64.89–65.66 ^a^. ^a^—Values expressed as %.	[22]
	1 *	Coconut sap	Malaysia (Jelai)	HPLC-RID (high-performance liquid chromatography coupled with refractive index detection)	Sample preparation: Coconut sap was diluted, (10×), filtered (0.45 µm), and further analyzed. Column: Merck LiChroCART^®^ Single bond NH_2_ column (250 × 4.6 mm; 5 µm). Mobile phase: Acetonitrile-water (80:20, *v*/*v*).	Three sugars (sucrose, fructose, glucose) were detected in coconut sap. Their respective values were 6.91 ^a^, 3.48 ^a^, and 2.53 ^a^. ^a^—Values expressed as %.	[36]
	3 *	Coconut sap (collected by two different methods) and coconut sugar	India (Kasaragod)	Phenol–sulphuric acid method (for total sugars content determination) Nelson-Somogyi’s method (for reducing sugars content quantification)	NR **	The total sugars content of the sap acquired traditionally, the sap obtained following a new “coconut-sap chiller method”, and the sugar produced from the latter was 9.20 ^a^, 16.2 ^a^, and 91.8 ^b^, respectively. For the reducing sugars content, the reported values were 1.24 ^a^ for the sap obtained conventionally, 0.68 ^a^ for the sap collected by the novel approach, and 4.69 ^b^ for sugar. ^a^—Values shown as g/100 mL; ^b^—Values shown as g/100 g.	[37]
	6 *	Coconut sugar and coconut syrup	Philippines (Makati)	GC–MS (gas chromatography-mass spectrometry)	NR **	The sugar composition of both coconut sugar and coconut syrup was evaluated for 6 months. For coconut sugar, the sucrose, glucose, fructose, and mannose levels ranged between 83.18–90.50 ^a^, 9.40–11.45 ^a^, 2.89–3.69 ^a^, and 0.51–3.90 ^a^, respectively. For coconut syrup, they varied between 35.85–38.96 ^a^, 10.74–14.03 ^a^, 15.39–15.57 ^a^, and 3.91–5.35 ^a^, respectively. ^a^—Values expressed in mg/100 g.	[38]
	4 *	Coconut sap (with and without preservative, i.e., limestone solution) and coconut sugar (with and without preservative)	Kemloko (Indonesia)	HPLC-RID (high-performance liquid chromatography coupled with refractive index detection)	Sample preparation: For each sample, 1 g was weighed, and then dissolved in 100 mL of distilled water. The mixture was filtered, and the solution was injected into the HPLC. system. Column: Aminex HPX-87C. Mobile phase: Water.	For the fresh sap to which no preservative was added, lower sucrose content (1.76 ^a^) and higher fructose (5.76 ^a^) and glucose (4.46 ^a^) contents were found compared to the sap with the preservative whose sucrose, fructose and glucose levels were 5.76 ^a^, 3.23 ^a^, and 2.25 ^a^, respectively. For coconut sugar, the sucrose content of that prepared with fresh coconut sap, but without adding a preservative, was lower (49.41 ^a^) than that of coconut sugar produced with the fresh coconut sap to which a preservative was added (49.41 vs. 57.05 ^a^). Their glucose (15.90 ^a^) and fructose (14.15 ^a^) levels were higher than those of the coconut sugar prepared from the fresh coconut sap to which a preservative was added; that is, glucose 6.97 ^a^ and fructose 5.45 ^a^. ^a^—Values expressed in %.	[32]
Phenolics	3 *	Coconut sap (collected by two different methods) and coconut sugar	India (Kasaragod)	Folin’s Ciocalteu method (for total phenolic content determination purposes) Ultrahigh performance liquid chromatography coupled with UHPLC-TQD-MS/MS (tandem quadrupole mass spectrometry) for the phenolic profile analysis	Phenolic profile: Sample preparation: The extraction of the individual phenolics was performed with 80% aqueous methanol (*v*/*v*). Thereafter, filtering the extracted sample was performed (0.2 μm). This sample was injected into the analytical system. Column: Waters UPLC BEH-C18 column (2.1 × 50 mm; 1.7 μm) protected by a Waters Vanguard BEH C-18 guard column (1.7 μm). Mobile phase: 0.1% formic acid in water 0.2% formic acid in methanol	The total phenolics content of the sap acquired traditionally, the sap obtained by a novel “coconut-sap chiller method”, and the sugar produced from the latter was 14.8 ^a^, 21.7 ^a^, and 47.2 ^b^, respectively. The following phenolic compounds were quantified in the coconut sap obtained by the traditional method: (i) vanillic acid (2.92 ^c^); (ii) syringic acid (1.80 ^c^); (iii) *trans*-cinnamic acid (0.636 ^c^); (iv) *p*-hydroxy benzoic acid (0.308 ^c^); (v) ferulic acid (0.302 ^c^); (vi) protocatechuic acid (0.182 ^c^); (vii) 2,4-dihydroxy benzoic acid (0.126 ^c^); (viii) gentisic acid (0.104 ^c^); (ix) gallic acid (0.073 ^c^); (x) *o*-coumaric acid (0.064 ^c^); (xi) rutin (0.043 ^c^); (xii) caffeic acid (0.042 ^c^); (xiii) salicylic acid (0.040 ^c^); (xiv) umbelliferone (0.030 ^c^); (xv) *p*-coumaric acid (0.008 ^c^). Regarding the sap collected by the new method, the identified phenolics were as follows: (i) vanillic acid (3.54 ^c^); (ii) *trans*-cinnamic acid (2.40 ^c^); (iii) *p*-hydroxy benzoic acid (0.963 ^c^); (iv) syringic acid (0.707 ^c^); (v) salicylic acid (0.477 ^c^); (vi) ferulic acid (0.246 ^c^); (vii) catechin (0.157 ^c^); (viii) quercetin (0.156 ^c^); (ix) hesperetin (0.116 ^c^); (x) myricetin (0.105 ^c^); (xi) caffeic acid (0.103 ^c^); (xii) rutin (0.078 ^c^); (xiii) protocatechuic acid (0.065 ^c^); (xiv) *o*-coumaric acid (0.062 ^c^); (xv) umbelliferone (0.056 ^c^); (xvi) gallic acid (0.044 ^c^); (xvii) *p*-coumaric acid (0.030 ^c^); (xviii) gentisic acid (0.026 ^c^); (xix) 2,4-dihydroxy benzoic acid (0.015 ^c^). In sugar: (i) vanillic acid (12.8 ^d^); (ii) benzoic acid (9.41 ^d^); (iii) *trans*-cinnamic acid (4.25 ^d^); (iv) catechin (2.37 ^d^); (v) *p*-hydroxy syringic acid (1.96 ^d^); (vi) *p*-coumaric acid (1.27 ^d^); (vii) ferulic acid (0.908 ^d^); (viii) *o*-coumaric acid (0.706 ^d^); (ix) salicylic acid (0.653 ^d^); (x) myricetin (0.390 ^d^); (xi) hesperetin (0.327 ^d^); (xii) quercetin (0.313 ^d^); (xiii) apigenin (0.230 ^d^); (xiv) protocatechuic acid (0.224 ^d^); (xv) gallic acid (0.203 ^d^); (xvi) rutin (0.192 ^d^); (xvii) gentisic acid (0.111 ^d^); (xviii) caffeic acid (0.109 ^d^); (xix) umbelliferone (0.078 ^d^); (xx) 2,4-dihydroxy benzoic acid (0.037 ^d^). ^a^—Values are mg of gallic acid equivalents (GAE)/100 mL; ^b^—Values are mg of GAE/100 g; ^c^—Values are mg/100 mL; ^d^—Values are mg/100 g.	[21]
Vitamins	1 *	Coconut sap	Malaysia (Jelai)	HPLC/UV-Vis (high-performance liquid chromatography coupled with ultraviolet-visible detection)	Sample preparation: Coconut sap was diluted (10x), filtered, and further analyzed. Column: Agilent Poroshell 120 EC column (100 × 4.6 mm; 4 μm). Mobile phase: Potassium dihydrogen phosphate buffer (pH 3.4, 50 mM).	Vitamins C, B1, B2, B3, B4, and B10 were all detected in coconut sap. Their levels were 116.19 ^a^, 4.33 ^a^, 0.084 ^a^, 1.88 ^a^, 0.53 ^a^, and 0.33 ^a^, respectively. ^a^—Values expressed as µg/mL.	[36]
	3 *	Coconut sap (collected by two different methods) and coconut sugar	India (Kasaragod)	6-Dichlorophenol-indophenol (DCPIP) method for vitamin C level measurements (ultrahigh performance liquid chromatography coupled to tandem quadrupole mass spectrometry) for the quantification of other vitamins	Sample preparation: Water-soluble vitamins: Samples were extracted with 10 mM ammonium acetate:methanol 50:50 (*v*/*v*) that contained 0.1% butylhydroxytoluene and centrifuged. Next, the supernatant was (0.2 μm) filtered and injected into the analytical system. Fat-soluble vitamins: The residue from the previously described extraction (please refer to “water-soluble vitamins”) was re-extracted with ethyl acetate that contained 0.1% butylhydroxytoluene, centrifuged and filtered (0.2 μm) before being injected into the analytical system. Column: Waters UPLC BEH-C18 column (2.1 × 50 mm; 1.7 μm) protected by a Waters Vanguard BEH C-18 guard column (1.7 μm). Mobile phase: Water-soluble vitamins: 0.1% formic acid in water- acetonitrile. Fat-soluble vitamins: Acetonitrile—0.2% formic acid in methanol.	The following vitamins were detected and quantified in the coconut sap obtained by the traditional method: (i) vitamin C—16.3 ^a^; (ii) B1—0.021 ^c^; (iii) B3—11.4 ^c^; (iv) B5—1.64 ^c^; (v) B6—1.32 ^c^; (vi) B7—0.095 ^c^; (vii) B9—0.031 ^c^; (viii) D2—0.028 ^c^; (ix) D3—0.062 ^c^; (x) E—2.94 ^c^; (xi) K1—0.601 ^c^; (xii) K2—0.428 ^c^. For the sap collected by a new “coconut-sap chiller method”, the vitamins and their respective levels were as follows: (i) vitamin C—19.6 ^a^; (ii) B1—0.068 ^c^; (iii) B3—14.9 ^c^; (iv) B5—3.99 ^c^; (v) B6—2.35 ^c^; (vi) B7—0.073 ^c^; (vii) B9—0.036 ^c^; (viii) D2—0.074 ^c^; (ix) D3—0.056 ^c^; (x) E—7.20 ^c^; (xi) K1—1.73 ^c^; (xii) K2—0.771 ^c^. Sugar contained (i) vitamin C—3.98 ^b^; (ii) B1—14.3 ^d^; (iii) B2—0.248 ^d^; (iv) B3—34.7 ^d^; (v) B5—2.53 ^d^; (vi) B6—101 ^d^; (vii) B7—2.51 ^d^; (viii) B9—0.260 ^d^; (ix) D2—0.171 ^d^; (x) D3—0.256 ^d^; (xi) E—19.6 ^d^; (xii) K1—7.35 ^d^; (xiii) K2—5.57 ^d^. ^a^—Values are given as mg/100 mL; ^b^—Values are given as mg/100 g; ^c^—Values are given as µg/100 mL; ^d^—Values are given as µg/100 g.	[21]
	6 *	Coconut sugar and coconut syrup	Philippines (Makati)	2,6-Dichloroindophenol titrimetric method	NR **	Vitamin C levels in both coconut sugar and coconut syrup were evaluated over 6 months and ranged from 16 to 44 ^a^ for the former, and from 19 to 30 ^a^ for the latter. ^a^—Values expressed as mg/100 g.	[31]
**Volatiles**							
Aliphatic/aromatic hydrocarbons, ketones, aldehydes, alcohols, esters, fatty acids, furans, pyrazines, pyrans and sulfur-containing compounds.	3 *	Coconut sap (fresh, clarified, and fermented)	India (Mandakalli)	GC–MS (gas chromatography-mass spectrometry)	Isolated volatiles: Volatile compounds were isolated by the SDE (simultaneous distillation-extraction) method with a Likens-Nikerson apparatus. The extractive solvent was dichloromethane. GC–MS: Column: Supelco-fused silica column SPB-1 (30 m × 0.32 mm; 0.25 µm) coated with polydimethyl siloxane. Gas carrier: Helium.	The following 21 compounds were identified in the fresh coconut sap: (i) palmitic acid (2024 ^a^); (ii) palmitoleic acid (1042 ^a^); (iii) ethyl lactate (560 ^a^); (iv) phenyl ethyl alcohol (357 ^a^); (v) 3-hydroxy-2-pentanone (236 ^a^); (vi) tetradecane (167 ^a^); (vii) farnesol (125.5 ^a^); (viii) 2-methyl tetrahydrofuran (105 ^a^); (ix) tetradecanone (104.5 ^a^); (x) tetradecanoic acid (94.0 ^a^); (xi) nonanoic acid (84.8 ^a^); (xii) dodecane (74.3 ^a^); (xiii) dodecanoic acid (52.6 ^a^); (xiv) hexanoic acid (49.8 ^a^); (xv) pentadecane (48.4 ^a^); (xvi) 2-hydroxy-3-pentanone (45.6 ^a^); xvii) nerolidol (44.9 ^a^); (xviii) hexadecane (37.2 ^a^); (xix) 1-hexanol (27.3 ^a^); (xx) hexadecanone (25.9 ^a^); (xxi) tridecanone (24.5 ^a^). For the clarified coconut sap, 13 compounds were identified, which were as follows: (i) palmitic acid (342 ^a^); (ii) ethyl lactate (300 ^a^); (iii) phenyl ethyl alcohol (195 ^a^); (iv) palmitoleic acid (141 ^a^); (v) 3-hydroxy-2-pentanone (75.9 ^a^); (vi) hexanoic acid (54.7 ^a^); (vii) tetradecane (46.9 ^a^); (viii) 2-methyl tetrahydrofuran (45.4 ^a^); (ix) dodecane (30.5 ^a^); (x) 1-hexanol (24.8 ^a^); (xi) pentadecane (21.8 ^a^); (xii) hexadecane (16.4 ^a^); (xiii) 2-hydroxy-3-pentanone (14.0 ^a^). In the fermented coconut sap, 11 compounds were identified, namely as follows: (i) palmitoleic acid (14,603 ^a^); (ii) isoamylalcohol (7467 ^a^); (iii) ethyl lactate (4636 ^a^); (iv) phenyl ethyl alcohol (4189 ^a^); (v) palmitic acid (2421 ^a^); (vi) dodecanoic acid (1084 ^a^); (vii) ethyl caprate (797 ^a^); (viii) ethyldodecanoate (709 ^a^); (ix) tetradecanoic acid (597 ^a^); (x) ethyl caprylate (503 ^a^); (xi) farnesol (224 ^a^). ^a^—Values expressed as µg/L.	[12]
	6 *	Coconut sap, coconut syrup and coconut sugar	Indonesia (Blitar)	Gas chromatography—mass spectrometry (GC–MS)	Isolated volatiles: Volatile compounds were isolated by a simultaneous distillation–extraction (SDE) method using a Likens–Nikerson apparatus. The extractive solvent was diethylether. GC–MS: Column: CBP-5 column (50 m). Gas carrier: Helium.	Five volatiles were isolated in the fresh coconut sap: (i) 2-butanol (60.26–68.37 ^a^); (ii) acetic acid (25.83–30.43 ^a^); (iii) 2-methylcyclohexane (0.66–6.39 ^a^); (iv) cyclohexyloctane (1.81–4.23 ^a^); (v) 1,4 dimethyl-6,1-butyl acetate (0.91–1.11 ^a^). For coconut syrup, the following occurred: (i) 2-butanol (45.35–51.02 ^a^); (ii) acetic acid (24.56–6.47 ^a^); (iii) dodecanoic acid (0.34–21.59 ^a^); (iv) 2-furan (1.97–6.73 ^a^); (v) cyclohexane (3.56–4.41 ^a^); (vi) 1,4 dimethyl-6,1-butyl acetate (0.40–10.26 ^a^); (vii) 4,6 dimethyl-5-cyclo-hexo pyrimidine (0–2.25 ^a^); (viii) 2,3 dimethylpirazine (0–0.77 ^a^). Finally for coconut sugar, the following compounds were identified: (i) acetic acid (21.54–35.05 ^a^); (ii) 2-butanol (29.98–31.23 ^a^); (iii) 1,4 dimethyl-6,1-butyl acetate (1.7–15.50 ^a^); (iv) N,N dimethyl 2-(diphenylmetoxi)-ethylamine (9.31–13.26 ^a^); (v) cyclohexyloctane (0.0–17.01 ^a^); (vi) dodecanoic acid (0.0–12.41 ^a^); (vii) methylpyrazine (1.46–1.81 ^a^). ^a^—Values expressed as %.	[37]
	1 *	Coconut sugar	Thailand (Samutsongkhram)	GC–MS (gas chromatography-mass spectrometry) GGO (gas chromatography-olfactometry)	Isolated volatiles: Volatile compounds were extracted three times with diethyl ether. The combined extract was left to concentrate in a Vigreux column. Then, the concentrated extract was subjected to high vacuum distillation and then concentrated, first in a Vigreux column and finally in a nitrogen flow. GC–MS: Column: Restek Stabilwax column (30 m × 0.25 mm; 0.25 µm) and an Agilent DB-5MS column (30 m × 0.25 mm; 0.25 µm). Gas carrier: Helium. Descriptive sensory analysis: The sensory evaluation panel included nine properly trained evaluators.	The following volatile compounds were identified in coconut sugar: (i) acetic acid ^a^; (ii) 2,3-pentanedione; (iii) acetoin; (iv) 2,5-dimethyl pyrazine; (v) 2,3-butanedione; (vi) methional; (vii) furfural; (viii) 5-methyl furfural; (ix) 2-furanmethanol; (x) 4-methyl-5H-furan-2-one; (xi) 5-methyl-2-furan methanol; (xii) benzylalcohol; (xiii) maltol [3-Hydroxy-2-methyl- 4H-pyran-4-one]; (xiv) Furaneol^®^ [2,5-dimethyl-4- hydroxy-3(2H)-furanone]; (xv) vanillin [4-Hydroxy-3- methoxybenzaldehyde]. The sweet, roasted, burnt, nutty, smoky, and caramel notes of coconut sugar were attributed mostly to pyrazine, furan, and pyran derivatives being present. Benzyl alcohol and vanillin also introduce sweet notes. In addition, acetoin, 2,3-pentanedione and 2,3-butanedione were found to be responsible for the buttery, cheesy, and creamy aromas. ^a^—Major component identified; values not reported.	[38]
	2 *	Coconut sugar	Thailand (Ampawa)	GC–MS (gas chromatography–mass spectrometry)	Isolated volatiles: Volatile compounds were extracted by SPME (solid-phase microextraction). GC–MS: Column: An Agilent DB-625 capillary column (30 m × 0.25 mm). Gas carrier: Helium.	The identified volatile compounds were as follows: (i) 2,3-diethyl-5-methyl pyrazine; (ii) 2,3-dimethyl pyrazine; (iii) 2,5- dimethyl pyrazine; (iv) 2-ethyl-3,5-dimethyl pyrazine; (v) 2-methyl pyrazine; (vi) ethyl pyrazine; (vii) 5-methyl furfural; (viii) furfural.	[12]
	2 *	Coconut sugar	Thailand (Samut Songkhram and Phetchaburi)	GC–MS (gas chromatography–mass spectrometry)	Isolated volatiles: Volatile compounds were isolated by means of headspace gas chromatography. GC–MS: Column: An Agilent DB wax-fused silica capillary column (60 m × 0.25 mm; 0.25 µm). Gas carrier: Helium.	The detected volatile components comprised the following: (i) ethanol (9.48–52.21 ^a^); (ii) 4-methanol (19.58–27.85 ^a^); (iii) acetaldehyde (<0.01–16.33 ^a^); (iv) 2-furanmethanol (<0.01–13.54 ^a^); (v) acetic acid (<0.01–11.98 ^a^); (vi) 1-hydroxy-2-propanone (<0.01–10.24 ^a^); (vii) acetone (2.63–9.98 ^a^); (viii) 2-ethyl-3,6-dimethyl pyrazine (<0.01–6.46 ^a^); (ix) 2-propanol (2.29–4.37 ^a^); (x) hexanoic acid (<0.01–2.93 ^a^); (xi) 3-methyl hexanal (<0.01–2.35 ^a^); (xii) 2-furaldehyde (<0.01–1.48 ^a^); (xiii) hydroxy-2-butanone (0.0–1.10 ^a^); (xiv) butanoic acid (<0.01–1.01 ^a^); (xv) 2-methyl propanal (0–0.91 ^a^); (xvi) 3-(methylthio)-propanal (<0.01 ^a^); (xvii) 2,3-butanedione (<0.01 ^a^); (xviii) 2-methyl-3-buten-2-ol (<0.01 ^a^); (xix) 2-methyl-1-propanol (<0.01 ^a^); (xx) 2-ethyl-5-methyl pyrazine (<0.01 ^a^); (xxi) 3-methyl-butanol (<0.01 ^a^); (xxii) 2-acetylfuran (<0.01 ^a^). ^a^—Values expressed as %.	[34]

* Total number of samples analyzed in the study; ** NR—Not reported.

**Table 3:** Detecting adulterants in coconut sap, sugar, and syrup.

Adulterants	Works						
	Samples			Methodology	Analytical Details	Principal Outcomes	Refs.
	No.	Kind	Origin				
**Cane and beet sugar**	21 *	Coconut sugar	NR **	^1^H NMR (proton nuclear magnetic resonance). ULPC-Q-TOF-MS (ultraperformance liquid chromatography quadrupole time-of-flight mass spectrometry). MRA (multivariate regression analysis).	^1^H NMR: Sample preparation: To identify polar minor metabolites, 500 mg of every sugar sample were dissolved in 1 mL of deuterium oxide to be vortexed. Then, a 600 μL aliquot was placed inside an NMR tube to be analyzed. To study the non-polar extracts, 1 mL of chloroform-*d* was added to 500 mg of all the coconut sugars. Then, suspensions were vortexed and centrifuged. Finally, a 600-μL aliquot of the supernatant was placed inside an NMR tube. Analysis: Spectra were recorded at 300 K. UPLC-Q-TOF-MS: Sample preparation: Of each sample, 1 g was dissolved in 20 mL of water and the solution was filtered (0.20 μm). A 2-μL aliquot of this filtrate was diluted (10x) to be then injected into the analytical system. Column: Waters HSS T3 C-18 column. Mobile phase: 15 mM acetic acid, 10 mM tributylamine, 5% (*v*/*v*) methanol-2-propanol.	Pyroglutamic acid has been identified as a unique marker for coconut sugar. Additionally, coconut sugars exhibited substantially higher levels of acetic, formic, lactic, and succinic acids than both cane and beet sugars. *Trans*-aconitic acid was shown to be a marker for cane sugar, as was betaine for beet sugar.	[26]
	11 *	Coconut sugar	Indonesia and unknown origin	Energy-dispersive X-ray fluorescence Soft independent modeling of class analogies (SIMCA)	Sample preparation: First, samples were ground, and pellets were prepared. To do so, 5 g of sugar needed to be mixed with 1 g of wax. Energy-dispersive X-ray fluorescence: The irradiation time (s) was 200 for Ca, Cl, Cu, Fe, K, P, and S, and 500 for Br, Rb, and Sr. Analytical parameters: LOQ *** (mg/Kg): 1.7 Br; 118.4 Ca; 78 Cl; 1.2 Cu; 4.6 Fe; 566 K; 171 P; 4.2 Rb; 1.19 Sr. Precision (%): 22 Br; 3.5 Ca; 2 Cl; 10.5 Cu; 6.5 Fe; 3 K; 6 P; 5 Rb; 8 Sr.	This research work established the mass fractions of Br, Ca, Cl, Cu, Fe, K, P, Rb, S, and Sr in the coconut, cane, and beet sugar samples. On average, all the aforementioned elements had significantly bigger mass fractions in coconut sugars than in cane and beet sugars.	[13,20,30]
Cane and corn sugar	109 *	Coconut sugar	Indonesia (Central Java)	δ^13^C IRMS (stable carbon isotope ratio mass spectrometry).	Sample preparation: First, 300 mg of all the coconut sugar samples were dissolved in 5 mL of deionized water in centrifuge tubes. Tubes were then immersed in warm water inside an ultrasonic bath (15 min). Then, solutions were (0.45 μm) filtered and a 10-µL aliquot was transferred to tin capsules, which were dried at 40 °C. Finally, capsules were crimped and subjected to double encapsulation prior to the analysis. Carbon isotope analysis: An isotope ratio mass spectrometer interfaced with an elemental analyzer (EA-IRMS) in the continuous flow mode for δ^13^C measured isotopes.	The genuine coconut sugar exhibited an average δ^13^C value of −25.6‰ ± 0.4‰. More positive δ^13^C values (>−24.8‰) indicate the addition of C4 sugar, i.e., cane or corn sugar/syrup. More negative δ^13^C values (<−26.4‰) should be related to the use of additives. On the whole, the authors propose a maximum acceptable δ^13^C value of −24.1‰ for authentic coconut sugars.	[25]

* Total number of samples analyzed in the study; ** NR—Not reported; *** LOQ—Limit of quantification.

**Table 4:** Detecting contaminants in coconut sap, sugar, and syrup.

Contaminants	Works						
	Samples			Methodology	Analytical Details	Principal Outcomes	Refs.
	No.	Kind	Origin				
**Insects**	NR **	Coconut sugar	NR **	NR **	NR **	The United Kingdom food safety authorities have reported the occurrence of insect fragments (500–800) in coconut sugar commercialized in the country.	[39]
**Microorganisms**	2 *	Coconut sap (collected by two different methods)	India (Kasaragod)	Serial dilution, plus, the spread plating method	Microbial analysis: The employed culture media were Nutrient agar, Martin Rose Bengal agar, Sabouraud Dextrose agar, and KenKnight & Munaiers agar, respectively, for bacteria, fungi, yeasts, and actinomycetes. Differing sample dilutions were plated in the respective medium and incubated at 28 °C. Bacteria and yeast colonies were scored as colony-forming units (CFU)/mL of sap after a 24-h incubation period, fungi/yeasts after 48–72 h, and those of actinomycetes after 1 week.	The conventionally collected coconut sap exhibited an extremely large number of bacteria and yeasts. In contrast, the sap collected by a new “coconut-sap chiller method” had a significantly lower level of microorganisms. The predominant populations were bacteria, namely those of the genus *Bacillus*. No actinomycetes growth was observed in either sample.	[21]
	4 *	Coconut sap (with and without preservative, i.e., limestone solution) and coconut sugar (with and without preservative)	Kemloko (Indonesia)	Total plate count	Microbial analysis: The procedure was performed in accordance with the “Indonesian National Standard for Microbe Contamination Test (Method 01-2897-1992)”. The employed culture medium was Nutrient agar.	The microbial counts of sap with and without a preservative were, respectively, “not countable/g” and 1.2 × 10^2^ colony-forming units (CFU)/g. The microbial counts of coconut sugar both with and without a preservative were, respectively, 1.2 × 10^2^ CFU/g and 3.6 × 10^2^ CFU/g.	[32]
	6 *	Coconut sugar and coconut syrup	Philippines (Makati)	Counts of: Aerobic plates. Coliforms. Moulds/yeasts. *Salmonella* sp.	Microbial analysis: An aerobic plate count was performed based on FDA-BAM-3; the coliform count was conducted based on FDA-BAM-4; the mould and yeast count was performed in accordance with FDA-BAM-18; the *Salmonella* sp. count was carried out following the FDA-BAM-5 procedure.	Coconut sugar exceeded the allowable limits for *Salmonella* sp. (/25 g; microorganism should not be detected) and coliform counts (should be <10 colony-forming units (CFU)/g). The values of the aerobic plate count, and the fungal and yeast counts, complied with legislation, i.e., they were below 10 CFU/g. With coconut syrup, the *Salmonella* sp. and coliform count values were in accordance with the stipulated criteria. The aerobic plate count exceeded the defined limit (<10–< 250 CFU/g).	[31]

* Total number of samples analyzed in the study; ** NR—Not reported.

## 3. Food Industry Applications and Sustainability Issues

For the application of coconut sugar in food and beverage industries, it is important to gain an understanding of the sap from which the sugar is processed. Coconut sap is a nutritious fluid enriched with sugars, calcium, phosphorous, iron minerals, and vitamins such as B complex and C [39,40]. It contains important phenolic compounds such as antioxidants and can be categorized as a low glycemic index (GI 35) food [40]. The nutritional composition of coconut sap is further described in Section 5.

Coconut sugar is made from the watery coconut sap found inside palm trees. It is prepared by concentrating inflorescence sap, which is popularly known as ‘neera’ in Kerala, India [23], and it is obtained by tapping the unopened coconut spadix. Coconut sugar is brown and contains 2–3% moisture. As this sugar is plant-based, natural, and minimally processed, it can be readily applied in many vegan diets as a healthier option [14,41].

The variation in coconut sugar manufacturing processes is extremely varied according to local, traditional, and indigenous knowledge [4]. These factors account for the vast variations in the appearance, taste, and flavor of the different coconut sugar types that can be found on the market [42].

Coconut sap is produced from palm trees all year-round and there is no specific season for tapping the spathe, however, the amount of sap produced from the trees changes with the season [40]. In the traditional method, the sap trickling from the cut surface is collected in an open earthen pot or bamboo sac, which is placed at the top of the palm for at least 8–12 h. Lime is then coated on the inner surface of the pot to prevent fermentation [43]. The sap collected by this method is oyster white in colour and emanates a strong odour with contamination from insects, ants, pollen, and dust particles [3]. The coco-sap chiller developed by Central Plantation Crops Research Institute (CPCRI) in India has helped to improve the quality of unfermented coconut sap, reduced the processing time, eliminated contaminants like ants, other insects, pollen, and dust particles, and enabled better product diversification and market perspectives [3]. A comparison of total sugars, reducing sugar, free amino acid, total flavonoids, and ferric reducing antioxidant power from coconut sap collected traditionally and those from coco-sap chillers are presented in Section 5. Similarly, a comparison of the water-soluble vitamins and fat-soluble vitamins is presented in table for the employed processing methods.

The Asian and Pacific Coconut Community describes how local operations are performed by small-scale cottage industries with coconut sap to yield molded coconut sugar. The traditional operation starts by collecting coconut sap from palms, which this is normally carried out twice a day, morning and evening [44]. The obtained coconut sap is then filtered through muslin cloth to remove ants, insects, and any other polluting elements. The filtered sap is placed inside cooking vessels. Sap concentrates by evaporating water to increase the sap concentration. This is achieved by boiling the filtered sap in cooking vessels for 3 h at 100–110 °C [44]. The resulting material then turns into a thick liquid. Upon boiling, foam forms that should be eliminated from vessels [44]. The usual procedure is to add a few drops of cooking oil or grated coconut to the resulting mash to prevent foam from excessively forming.

This mash is heated for another hour and is occasionally stirred. To prevent sugars from caramelizing, the must be heated slowly [7]. When the mash is very thick and suitable for molding, cooking vessels are lifted from stoves and cooled to 60 °C. The cooled mash is poured inside clean half coconut shells or bamboo vessels to be cooled and to set [45].

The processing technique influences nutritional and health benefits, as described in the previous section. To ensure product quality, the collected sap is tested for its acidity. This is crucial because, if sap is fermented, it is not suitable for brown coconut sugar manufacturing purposes.

Given its high sucrose content, during its storage, coconut sugar displays caking properties. So, it is essential to add an anticaking agent like tricalcium phosphate (TCP) for it to remain stable during food applications. TCP covers the coconut sugar powder surface and its hygroscopicity significantly diminishes, which improves its flowability [45].

Processing coconut sap into sugar syrup has been investigated following several alternative processing techniques. The coconut sugar syrup obtained from the rotary evaporation method has a better nutritional value than the microwave heating and open heating methods [22]. The non-enzymatic browning that results from Maillard reactions (MR) is enhanced when cooking sap at higher temperatures for a long period, which gives the preferred dark-colored coconut sugar as an ingredient, but only for traditional dishes [46]. Rotary evaporation is fast and gentle and performed at a lower temperature. All this results in evaporation with less thermal decomposition [47,48,49]. The rotary evaporation method is the alternative processing method that the sugar processing industry applies to produce coconut sugar. It operates in a 250-mbar vacuum at 60°C. It results in improved physico-chemical qualities, minimum input energy, and shorter processing times [43].

The employed processing method influences the antioxidant properties and vitamin contents of coconut sugar syrup. It allows coconut sugar production in a minimum time period, but with high vitamin and antioxidant contents [50]. The coconut sugar syrup produced by at 60 °C rotary evaporator (RE-60) shows significantly lower antioxidant activities (DPPPH, ABTS, FRAP, and TPC) values (*p* ≤ 0.05) than that generated by other techniques (open-heat evaporation, microwave, etc.). What this suggests is that the coconut sugar syrup that is produced at a lower temperature (60 °C) in vacuum exhibits significantly different and lesser antioxidant activities than all the other samples generated by distinct evaporation techniques [11,45].

Employing coconut sugar syrup with vast amounts of antioxidants is a promising food production ingredient. Former research works have observed how coconut sugar with larger quantities of vitamins and minerals and that perform more antioxidant activities can be used as an alternative natural sugar with improved chemical properties [3,51].

The work by Saputro [52] reveals the use of low-glycaemic-index (GI) sugar, such as (coconut sugar), to produce plain chocolate. They demonstrated that it was more nutritious as a sugar containing more anti-carcinogenic compounds, antioxidants, and minerals than commercial chocolates made with sugarcane sugar or sugar palm. Moreover, if coconut sugar can be employed as an ingredient, it is able to generate more antioxidant activity if food is processed at high temperatures. Very important compounds such as pyroglutamic acid or hydroxymethylfurfural (HMF) form when heated [53].

Coconut blossom sugar is organic with a caramel aroma and has been the target of adulteration and fraud [6,26]. A recent study identified minor metabolites, such as chemical markers for coconut blossom sugar, by profiling these metabolites, which helped to detect adulterations in products. Bachmann et al. [26] were unable to detect HMF in all the samples. However, pyroglutamic acid was employed at a comparatively high concentration, which exceeded other unambiguous metabolites in coconut sugar like inositol or shikimic acid in coconut sugar [26]. Henceforth, HMF is an apparently suitable marker metabolite for coconut sugar. The distinct metabolic profiles of coconut blossom sugar can be better investigated and identified by combining LC-MS and NMR [54].

**Figure 1:**
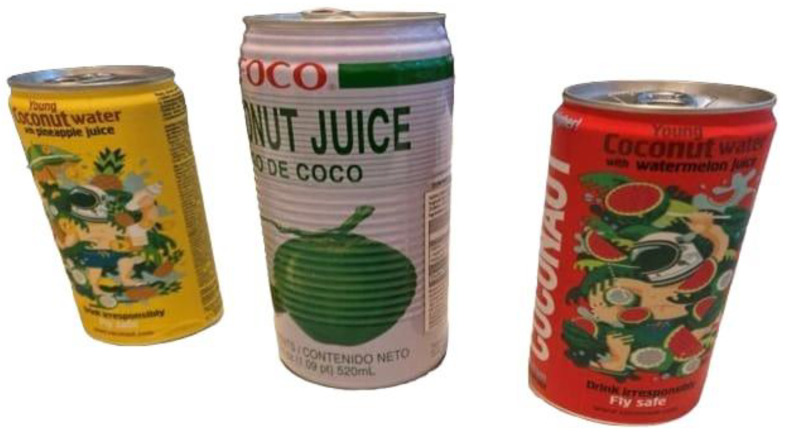
Coconut sap as beverages, bought from a local ethnic shop in Rovaniemi, Finland. (Photo credit: ©Dele Raheem, July 2022).

Coconut sap as a natural non-alcoholic beverage has high demand as an instant thirst quencher. In India, tapping coconut sap has improved the income of farmers and generated employment. Export of the sap is extensively carried out to countries like Canada, South Korea, USA, Norway, France, Japan, Australia, and the Middle East [55]. Coconut water and juice from coconut sap are commercially canned as beverages in Thailand and exported as ‘functional food‘ with health benefits (see Figure 1). These beverages are flavoured with tropical fruits such as watermelon and pineapple. Globally, the beverages industry was forecasted to reach $1.9 trillion in 2021 and continue to grow at a compounded annual growth rate (CAGR) of 3% [56].

Numerous organic food and drink firms increasingly employ natural alternative sweeteners such as coconut sugar to substitute refined sugars. Coconut sugar is employed thanks to its ecological credentials and nutritional properties. It has many widespread applications in food and beverage industries to prepare bakery products like chocolate (plain chocolate and drinking chocolate), cake, cookies, and brownies. It can be added to juice, tea, or any beverage as a sweetener, and can be employed as a seasoning agent. Adding coconut sugar to several food applications as a healthy option is well justified because it contains important nutrients like vitamins E and C, minerals like zinc, iron, potassium, and phosphorus, and phytonutrients like anthocyanidins, flavonoids, polyphenols, and antioxidants [3,20,35]. This kind of sugar also contains a significant amount of inulin (4.7 g 100 g^−1^), required for generating short-chain fatty acids like acetate, butyrate, and propionate [15].

The sugar obtained from the sap of palm trees, which includes coconut sap, is utilized mainly in desserts, sweet soy sauce, and beverages, and also in many other traditional foods. This is especially due to its appreciated and accepted taste, color, and flavor when producing drinks and foods [57,58,59,60]. Apriyantono [61] indicated that using palm sugar as a soybean sauce sweetener strongly impacts soy sauce flavor because over 70 volatile compounds are present. Employing palm sugar as a potential natural sweetener also impacts cookies’ color, textural properties, and flavor [62], which lends this sugar to being a potential natural sweetener.

Pure sucrose is the most widely used sugar as food sweetener. However, coconut sugar is reported to offer health benefits thanks to its lower GI value. The GI values previously obtained from coconut sugar [63,64] are below the GI values for pure sucrose, i.e., refined cane sugar [65]. Pure sucrose is the most commonly employed sugar as food sweetener. During baking operations, and as another research work reveals, palm sap sugar-sweetened bread has a lower GI value than cane sugar-sweetened bread [66]. Moreover, Ref. [66] report that the palm sugars–corn starch mixture brings about a slow digestion rate and, consequently, lower GI values than those made with refined cane sugars. Coconut sugar displays good quality and possesses a high nutritive value if it is processed from hygienic non-fermented sap; however, if poor-quality neera is employed, its crystallization involves having to add several additives and chemicals (e.g., starch and gluten, and adding sugars from C4 plants, palm, or coconut oil). During the manufacturing process, coconut quarters are added to avoid overboiling sap [1].

Regarding sustainability and traceability issues, organic certification comes over as a quality standard that helps to increase coconut sugar’s credibility in the European market. In most cases, coconut sugar exporters are from developing countries. They should consider not only certification, but also natural and organic trends [7]. Consumers will also show an interest in the story behind sustainable production. Export traders can advertise that small farmers traditionally produce coconut blossom sugar, palm trees organically grow mixed with other crops, and sugar has very low fructose contents and lower GI values than traditional refined beet or cane sugar [7]. 

As the interest in climate change is growing, individual and planetary healthy coconut-based ecosystems offer excellent possibilities to enhance carbon sequestration with crop combinations that involve a range of plants, which include vine, food crops, tubers, and tree crops. For climate-change adaptation intentions, annual intercrops planted under coconuts can be managed to achieve optimum benefits for the whole system. A holistic approach that focuses on the whole system’s overall productivity and sustainability, and not on palms alone, is necessary to make coconut-based agroecosystems resilient to climate change [67]. The demand for natural products is expected to grow, and employing alternative sweeteners, such as coconut syrup and sugar, will increase.

## 4. Safety and Quality Conditions for Control of Palm Sap Sugar Products

Both palm sap sugar (PSS) and sweet sap are alternative sweeteners prepared from the sap and nectar tapped from the flowers of several palm tree species. For example, palmyra palm (*Borassus flabellifer*), nipa palm (*Nypa fruticans* Wurmb), sugar palm (*Arenga pinnata*) and coconut palm (*Cocos nucifera*). They have the potential to be incorporated into food products as substitutes for sucrose [67]. This sweet sap can be consumed fresh, processed as sugar or syrup, or be fermented as vinegar or an alcohol beverage [68]. This sugar is commonly used in many traditional foods in southeast and southern Asia, and plays a vital role in the color-flavor development of distinct food products [57,58,59]. One major palm sugar exporting country is Indonesia. Based on the most recent data, the exports of products made with palm sugar or coconut sap came to 36.5 thousand tons, valued at US$49.3 million in 2019 [69]. These products destined for export must comply with the food legislation of the country of destination, such as, the European countries (EFSA) or United States (FDA). 

The world’s PSS business is expected to reach a total of 1.7 billion dollars in 2027, and is currently 630 million dollars [70]. This increase might be due to its potential to be incorporated into food products as a substitute for sucrose [71]. This product is often employed in many traditional foods in Asia, where it plays an important role in the color–flavor development of different food products [57,58,59]. Unlike other natural sweeteners, its production is located in a limited number of countries or in a certain geographical area; for example, agave is produced mainly in Mexico and maple syrup in Canada and the USA. However, PSS is produced in southeast and southern Asia, and the mainly producing countries are the Philippines, Thailand, and Indonesia. 

Around the world, there are more than 3000 different types of palm trees, but only five are economically important. They offer good sugar palm production yields for any of its different products. They are as follows: date palm (*Phoenix dactylifera*), betel nut palm (*Areca catechu*), African oil palm (*Elaeis guineensis*), coconut (*Cocos nucifera*), and pejibaye (*Bactris gasipaes*) [72]. Other authors include more palm species [73], such as the following:

- Coconut palm sugar (*Cocos nucifera*). It grows in coastal tropical regions of the Indian and Pacific oceans. This sugar is generated from blossoms and is often known as coconut blossom sugar.
- Date palm has two varieties (*Phoenix sylvestris* and *Phoenix dactylifera*). They can be found in Asia and the Middle East, respectively. Date palms are grown mostly for their fruit: dates.
- Palmyra palm (*Borassus genus*). It grows in the African continent and in Asia and New Guinea. It is used for making hats, hatching, writing materials, and some food products. Obviously, its wood is employed. Palm sugar is generated from the sap (called ‘toddy’) of tree flowers.
- Nipa palm (*Nypa fruticans*). It is found in tropical and coastal regions of the Pacific and Indian Oceans. It lies particularity in its favored biome: mangroves. It is the only palm tree that partially grows underwater. Its tap is rich in sugar, and it is employed to produce palm sugar.
- Sugar palm (*Arenga pinnata*) is native to tropical and coastal regions in Asia. It is grown mostly in Indonesia and China. The sap used to generate palm sugar is called ‘gur’ and ‘gula aren’ in India and Indonesia, respectively.

Nevertheless, other authors acknowledge 40 palm species, the tapping of which is either destructive or non-destructive. Non-destructive exploitation with, for instance, *Phoenix canariensis* on the Canary Islands (Spain) results in sustainable harvests during palms’ lifetime [68].

Special attention is paid to harvest the sap tapping of *Phoenix canariensis* for its sugary sap on the La Gomera Isle (Canary Islands). It is one of the most relevant cases of sustainable native flora use. It supplies one of the best-known ethnobotany examples on the Canary Islands and is not only a major tourist attraction for visitors, but also an important local farming activity [68].

PSS is produced with the sap/nectar that is tapped from the flowers of several palm tree species. Knowledge about the physico-chemical properties of this sugar should be known if a high-quality product is to be obtained. PSS’ physico-chemical characteristics are affected by its raw materials (sap/nectar) and processing techniques [37,52,73]. Further, the form that sugars come in (syrup, coarse/powder, solid) also determines its properties. Coconut sap, the natural and sweet exudate from tapped unopened coconut spathes or inflorescences (*Cocos nucifera* Lin.), is one of the major primary coconut pro- ducts used for many food uses. It can be processed as natural and nutritious food products, such as coconut granulated brown sugar, concentrate, juice, and vinegar. Processes involve easy-to-follow procedures that require a few simple tools and equipment.

Coconut sap juice is a healthy pasteurized beverage, and coconut sap concentrate is a thick, free-flowing syrup. Both can be considered functional foods for consumers and the food industry.

The inflorescence in good stands of coconut trees can produce an average of 2 L of sap per tree a day [74]. An average yield of 1 kg of sugar can be obtained from four coconut trees every day.

Under adequate production conditions, coconut trees’ inflorescence can produce a mean yield of 2 L of sap per tree every day. So, the yield of four coconut trees per day can produce 1 kg of sugar. However, as both the sugar content and production of sap depend on trees’ location and their variety, nutrition, the season, tapping time, and the system, these conditioning factors also can impact organoleptic and microbiological characteristics.

Some authors have followed different preservation techniques for bottling palm sap; although all probes failed, these authors consider it crucial to understand the biochemical composition, fermentation chemistry, and existing preservation methods [75].

Transforming coconut sap into sugar granules is simple and requires basic equipment, hence, it is appropriate for and best adapted to farms or medium-sized enterprises. It is a good source of immediate income for coconut farmers, and demand is growing on both local and international markets [76].

Engineering the palm sugar production process poses several problems if sap is not immediately cooked after it is removed from palm trees, which results in a lower pH. A drop in pH impacts the produced palm sugar’s quality. To obtain a higher product conversion factor value, engineering the production process by adding plant extracts to prevent “gait” is feasible [74], and the palm sugar packaging design is more appealing and varied. Packaging is designed by prioritizing practical, economical, and hygienic aspects, without burdening producers in production terms and consumers in price terms. One alternative for PSS with a high quality (soluble solids content 16 degrees brix and pH 4.7) has been presented with the addition of some preservatives such as citric acid (0.09%) and nisin (10 ppm) [76]. The quality standard in the Philippines includes quality norms to not only produce PSS, but also to obtain a product of standardized quality and well-defined organoleptic and microbiological parameters.

### Coconut Sap Sugar Production in the Philippines 

**Figure 2:**
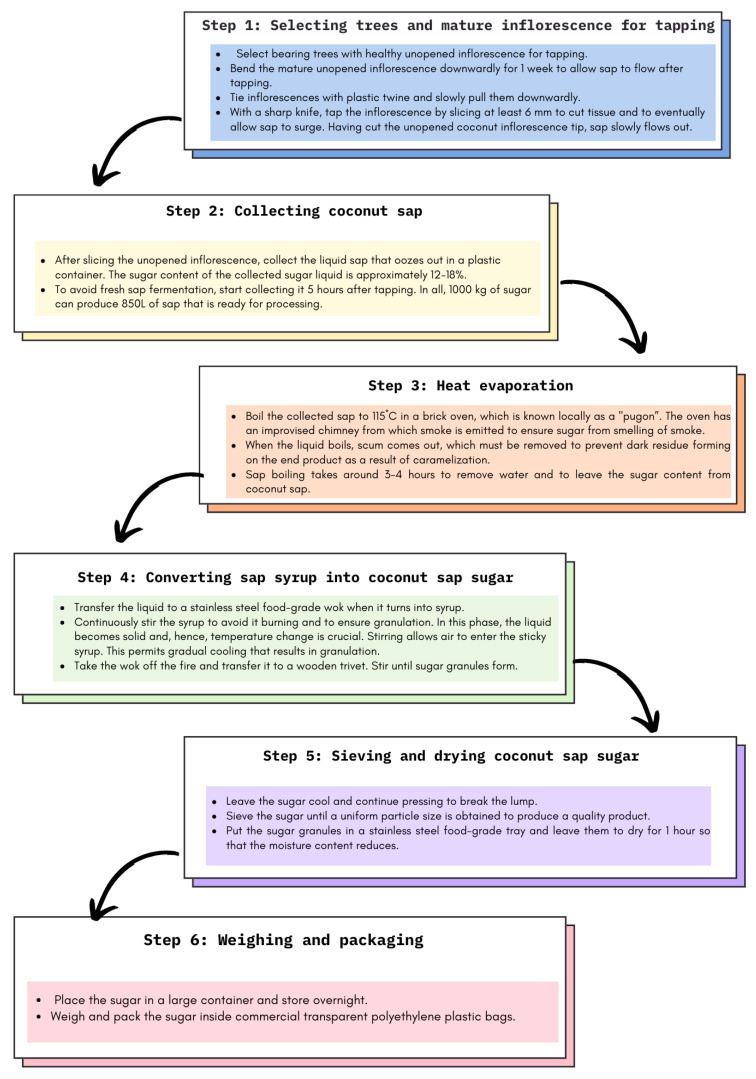
Recommendations for coconut sap sugar production in the Philippines. Adapted from Refs. [17,70,74,77].

General considerations: farm-level technology to produce a high-value production product from coconut inflorescence sap (see Table 5). It is simple, farm-level technology that involves a natural heat evaporation process that converts liquid sap into a solid form of sugar granules without having to resort to complicated and costly machinery or equipment (Figure 2). 

## 5. Nutritional Profile and Health Impacts

Despite its expensive price, coconut sugar is considered by several authors to be one of the greatest natural sweeteners as it offers a number of health advantages [14]. Table 6, Table 7 and Table 8 list the biochemical properties, vitamins, and other nutritional components of the sugar obtained from coconut inflorescence sap.

Vitamins C and E, minerals including zinc, iron, potassium, and phosphorus, and phytonutrients like antioxidants, flavonoids, anthocyanidins, and polyphenols, are all present in coconut sugar [21]. Additionally, inulin comes in a substantial quantity (4.7 g, 100 g^−1^) in coconut sap sugar. It is necessary for the synthesis of short-chain fatty acids acetate, butyrate, and propionate [31]. Coconut sugar and syrup (the latter contains dietary fiber and fermentable inulin) are truly promising functional foods and are converted into short-chain fatty acids [31]. As coconut sugar has a sweetening potential that is comparable to saccharose, it is utilized as an alternative sweetener for making confections, drinks, pastries, and other gastronomic delicacies [78]. According to Trinidad et al. [63], coconut sugar has a low GI that falls within the 35–54 range per serving. Low GI diets lower the likelihood of developing certain chronic diseases like type II diabetes. Compared to other sugars, coconut sugar has nutritional superiority. When cane, palm, and coconut sugars, sorbitol, and other sweeteners are blended with wheat flour, sorbitol possesses the best starch digestibility—with an almost identical digestibility for palm or coconut sugars [66]

Compared to the majority of other commercially available sugars, coconut sugar is certainly a healthy sweetener. It is processed by evaporating sap—which requires considerable labor and resources when collected from trees—that is then transported, stored, and processed. Therefore, the manufacture cost is higher than for cane sugar. People are willing to pay high prices for it given its nutritional value and low GI. However, one bottleneck is the lack of knowledge about its health advantages. Natural coconut sugar and other biproducts are produced hygienically as a result of scientific developments in sap collecting and processing, which have occurred in some major producing nations, including India, in the last few years [14]. 

**Table 6:** Coconut inflorescence sap obtained with a Cocosap chiller (conventional method) and coconut sugar were studied for their biochemical components and ferric-reducing antioxidant power [14].

Biochemical Characteristics	Coconut Inflorescence Sap Obtained by the Cocosap Chiller Method (100 mL)	Traditionally Collected Sap (100 mL)	Coconut Inflorescence Sap Sugar (100 g)
Total sugars (g)	16.20 ± 0.33	9.20 ± 0.97	91.8 ± 1.01
Reducing sugars (g)	0.68 ± 0.01	1.24 ± 0.87	4.69 ± 4.60
Free amino acids (g)	1.03 ± 0.10	0.413 ± 0.09	3.05 ± 0.07
Total phenolic content (mg gallic acid equivalent)	21.7 ± 0.48	14.8 ± 1.03	3.05 ± 0.07
Total flavonoids (mg catechin equivalent)	0.817 ± 0.19	0.177 ± 0.02	4.76 ± 1.21
Ferric-reducing antioxidant power (mg of ascorbic acid equivalent)	14.8 ± 0.21	8.34 ± 0.83	22.9 ± 4.12

**Table 7:** Vitamin composition of coconut inflorescence sap obtained with a Cocosap chiller (conventional method) and coconut sugar [14].

Biochemical Characteristics	Coconut Inflorescence Sap Obtained by the Cocosap Chiller Method (100 mL)	Traditionally Collected Sap (100 mL)	Coconut Inflorescence Sap Sugar (100 g)
Water-soluble vitamins			
Vitamin C (mg)	19.6 ± 0.95	16.3 ± 0.76	3.98 ± 1.12
Thiamine (µg)	0.07 ± 0.02	0.02 ± 0.00	14.3 ± 1.16
Niacin (µg)	14.9 ± 2.80	11.4 ± 0.7	34.7 ± 2.1
Pyridoxine (µg)	2.35 ± 0.01	1.32 ± 0.12	101 ± 0.3
Pantothenic acid (µg)	3.99 ± 0.08	1.64 ± 0.11	2.53 ± 0.2
Biotin (µg)	0.07 ± 0.01	0.09 ± 0.01	2.51 ± 0.7
Folic acid (µg)	0.036 ± 0.01	0.031 ± 0.00	0.26 ± 0.07
Riboflavin (µg)	-	-	0.25 ± 0.02
Fat-soluble vitamins			
Cholecalciferol (µg)	0.056 ± 0.00	0.062 ± 0.00	0.256 ± 0.02
Ergocalciferol (µg)	0.074 ± 0.01	0.028 ± 0.00	0.171 ± 0.02
Tocopherol (µg)	7.20 ± 0.93	2.94 ± 0.46	19.6 ± 3.5
Vitamin K1 (µg)	1.73 ± 0.19	0.601 ± 0.09	7.35 ± 0.95
Vitamin K2 (µg)	0.771 ± 0.12	0.428 ± 0.12	5.57 ± 0.61

**Table 8:** Nutritional profile of coconut sugar made from inflorescence sap on a double-jacketed cooker and a modified conventional processing technique. (The results should be interpreted in light of the biochemical characteristics of coconut sugar, as listed in Table 6 and Table 7).

Biochemical Components	Content
Protein (g/100 g)	2.6
Dietary fiber (g/100 g)	3.1
Electrolytes (mg/100 g)	
Sodium	568
Potassium	1002
Microminerals (mg/100 g)	
Iron	2.2
Zinc	2.1
Essential amino acids (mg/100 g)	
Valine	40.68
Threonine	45.81
Leucine	16.01
Lysine	136.5
Methionine	54.55
Histidine	3.48
Phenylalanine	57.66
Tyrosine	5.68

## 6. Conclusions

The global drive toward better individual and environmental health warrants the need for better knowledge about what we produce and consume. Sweeteners are important food ingredients to formulate edible food products, and for health and sustainability. This review summarizes the micro- and macrocomponents isolated from coconut sugar, sap, and syrup, the chemical components of these natural sugars, and their physico-chemical, microbiological, and antioxidant characteristics. A better understanding of these components reveals the health-giving properties of coconut as a plant-based sugar, despite the associated costs of taking coconut-based foods to consumers. Hence, it is important that food industries respond to the demand of health-conscious consumers by incorporating coconut sugar, sap, and syrup into food products. Some shortcomings in this review, which can be addressed in the future, are the need to consider personal dietary preference of coconut sugar in food products, sustainability issues by more rigorous studies, and to study the role of coconut trees and carbon sinks, including life cycle assessments (LCAs).

## Figures and Tables

**Table 5:** Reference values for palm sap sugar products. Adapted from Ref. [74].

Physico-Chemical Properties	Reference Values
Color	light yellow to dark brown
Odor	free of burnt odor
Taste	free of burnt taste
Moisture Content (%)	<4.0
Glucose Content	2.8–3
Fructose Content	1.0–4.0
Sucrose	78.0–89.0
Ash	<2.4
**Microbiological properties**	
*Salmonella*	Negative
*E. coli*	Negative
Coliform count	<10 ufc/g
Total Plate Count	<10 ufc/g
Molds and Yeasts	<10 ufc/g

## Data Availability

Not applicable.
